# Effects of Different Management Practices on Ramet System Dynamics in Moso Bamboo (*Phyllostachys edulis*) Forests, China

**DOI:** 10.3390/plants14121835

**Published:** 2025-06-14

**Authors:** Guibin Gao, Xing Wen, Jinfang Qian, Yiji Huang, Zhizhuang Wu, Hao Zhong, Yanhong Pan, Xiaoping Zhang

**Affiliations:** 1 China National Bamboo Research Center, Key Laboratory of State Forestry and Grassland Administration on Bamboo Forest Ecology and Resource Utilization, Hangzhou 310012, China; anshu998@caf.ac.cn (G.G.); wenxing202202@163.com (X.W.); wzzcaf@126.com (Z.W.); zhonghao0726@163.com (H.Z.); zhukan2004@163.com (Y.P.); 2 National Long-Term Observation and Research Station for Forest Ecosystem in Hang-Zhou-Jiaxing-Huzhou, Plain, Hangzhou 310012, China; 3 Changxing County Natural Resources and Planning Bureau, Huzhou 313100, China; 15869175672@163.com; 4 Shuikou Township Forestry Technology Extension Station, Changxing County, Huzhou 313108, China; zxpyg2016@126.com

**Keywords:** ramet system, Moso bamboo, bamboo forests management, rhizome, bud bank, branch

## Abstract

Examining the ramet system in bamboo forests can provide an important theoretical basis for strategic management. Moso bamboo is an economically important species in China, and implementing the correct management measures can play a key role in improving bamboo productivity. However, the dynamics of the Moso bamboo ramet system under timber vs. shoot forest management remain underexplored. In this study, we investigated the underground rhizome growth, bud bank structures, branch growth, and distribution patterns of bamboo ramet systems in the two main bamboo cultivation types. Shoot forest ramet systems exhibited stable early-stage rhizome renewal but instability in later stages, characterized by thin, elongated rhizomes. The opposite was observed in the timber forests. The underground bud bank of the ramet system in the shoot forest had a strong renewal ability with stable lateral bud input. However, shoot harvesting disturbed the bud bank balance. The lateral bud input in the timber forest was unstable, with the lateral buds being prone to death. The variation range and quantity of branch types in the ramet system in the shoot forest were greater than those in the timber forest. The number of branches in different parts of the ramet system was in the order of rhizome tip (RT) > middle of rhizome (RM) > rhizome base (RB). The range of variation was greater in the shoot forest. Different management methods led to growth differences in the examined bamboo ramet systems. Bamboo forest management resulted in a correlation between bud banks and ramet system renewal. Operations such as bamboo shoot harvesting significantly impacted branch growth and distribution. These findings not only provide a better understanding of the growth and management strategy of bamboo ramet systems worldwide but also provide a universal theoretical reference for the sustainable management of bamboo forests in other countries.

## 1. Introduction

The ramet systems of clonal plants play a key role in maintaining population stability and expansion [1,2]. The ramet systems of some grassland or desert clonal plants can adjust their growth rate and biomass allocation to maintain population stability under different environmental conditions [3,4,5]. The responses of clonal plant ramet systems to environmental changes have been extensively studied. The ramet systems of clonal plants can adjust the number of branches and amount of rhizome material transfer through clonal integration according to the light intensity or under waterlogging and saline–alkaline stress to maximize photosynthetic efficiency and enhance their adaptability to the alternating environments [6,7,8,9].

Bamboo ramet systems remain understudied, particularly in managed ecosystems. Under heterogeneous environmental conditions, changes in soil nutrients, water, and growth environments, such as light levels in bamboo forests, can lead to changes in the structure and function of ramets or certain bamboo clonal fragments in the ramet system [10,11,12]. However, the bamboo forest ramet system represents a unique structural unit with independent and interacting parts, and it has not been well explored. Some studies on ramet systems have been conducted in artificially controlled environments, such as with potted plants [13], or focused on the impacts of a single factor on bamboo ramet systems. The growth characteristics of ramet systems have been examined in intensively mulched bamboo forests [14]. However, to date, most of these studies have primarily focused on one type of bamboo forest management, making it difficult to fully understand the effects of bamboo forest management on bamboo ramet systems.

Moso bamboo (*Phyllostachys edulis*) is the bamboo species with the largest distribution area and most extensive use in China. It is widely planted in areas south of the Yangtze River in China [15]. Moso bamboo has considerable value for research and use. Moso bamboo forests can help in conserving soil and water, purifying air, and regulating the climate, playing a key role in maintaining regional ecological balance [16]. The Moso bamboo industry encompasses many fields, including bamboo processing, shoot consumption, and handicraft manufacturing. It is a vital economic resource for many mountain farmers and is of considerable importance for promoting local economic development [17]. Moso bamboo forests are widely cultivated using diverse management practices. Timber and shoot forests are the two Moso bamboo forest cultivation and management types. Timber forests generally have stable bamboo production. Meanwhile, shoot forests are used for high-efficiency bamboo shoot harvesting. However, at present, the growth characteristics of the ramet system under different management practices are unclear.

Based on the strong ecological adaptability of bamboo, the structure and function of bamboo ramet systems under different management conditions may undergo tremendous changes. In this study, comparative investigations of timber and shoot forests revealed how morphological plasticity influences bamboo ramet system underground rhizomes in the context of artificial management. By analyzing the changes in the bud bank of the ramet system from input to output, we explored the self-maintenance and renewal characteristics of bamboo ramet systems. By analyzing factors such as management methods and differences in the growth activities of different parts of underground rhizomes, we have deepened our understanding of the response laws of the branch growth and distribution patterns of bamboo ramet systems to the interactions among them. Our findings can provide a reference for efficiently enhancing the productivity and sustainability of bamboo forest ramet systems and offer a theoretical basis for implementing strategic management practices in bamboo forests.

## 2. Results

### 2.1. Growth of Underground Rhizomes of Bamboo Ramet Systems

**Figure 1:**
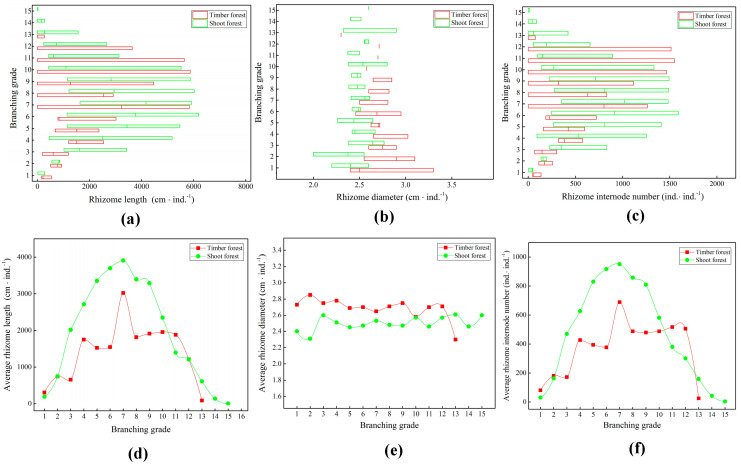
Differences in morphology of underground rhizomes at different branch grades of ramet systems. (**a**) Quantity distribution of rhizome length; (**b**) quantity distribution of rhizome diameter; (**c**) distribution of number of rhizome nodes; (**d**) change trend of average rhizome length; (**e**) change trend of average rhizome diameter; (**f**) change trend of average number of rhizome nodes.

As shown in Figure 1, in the shoot forest, the variation ranges for rhizome length (Figure 1a) and the number of rhizome nodes (Figure 1c) of the ramet system at low branch grades were relatively high. Meanwhile, those at high branch grades were relatively low. This suggested stable early-stage rhizome renewal in shoot forests, with instability emerging as the rhizomes aged. Contrary to the shoot forest, the variation in rhizome length (Figure 1a) and number of rhizome nodes (Figure 1c) of the ramet system in the timber forest at low branch grades were relatively low. Meanwhile, those at high branch grades were relatively high. This indicates that the underground rhizome renewal growth of the ramet system in timber forests is relatively unstable. However, with the continuous aging, death, and disintegration of the ramet system branches, the manifestation of this unstable trait tends to weaken. The range of variation in the diameter of the underground bamboo rhizomes of the ramet system in the timber forest was greater than that in the shoot forest (Figure 1b). This indicates that the heterogeneity of the nutrient environment for the growth of bamboo rhizome branches in the ramet system of the timber forest was relatively high.

The average length (Figure 1d) and the number of rhizome nodes (Figure 1f) in the shoot forest showed a single-peak trend, exceeding timber forests at most grades. The average rhizome diameter (Figure 1e) of the bamboo rhizome branches of the ramet systems in the shoot and timber forests did not change significantly with an increase in branch grade. The average rhizome diameter of the ramet system in the timber forest was 2.76 ± 0.19 cm, which was higher than 2.49 ± 0.08 cm in the shoot forest. The bamboo rhizome branches of the ramet system in the shoot forest were thin and long, while those of the timber forest were thick and short. This indicates that the survival strategy of the ramet system in the timber forest tended to consolidate itself. Meanwhile, in the shoot forest, the ramet system tended to expand vigorously in all directions.

### 2.2. Structure of Underground Bud Banks of Bamboo Ramet Systems

**Figure 2:**
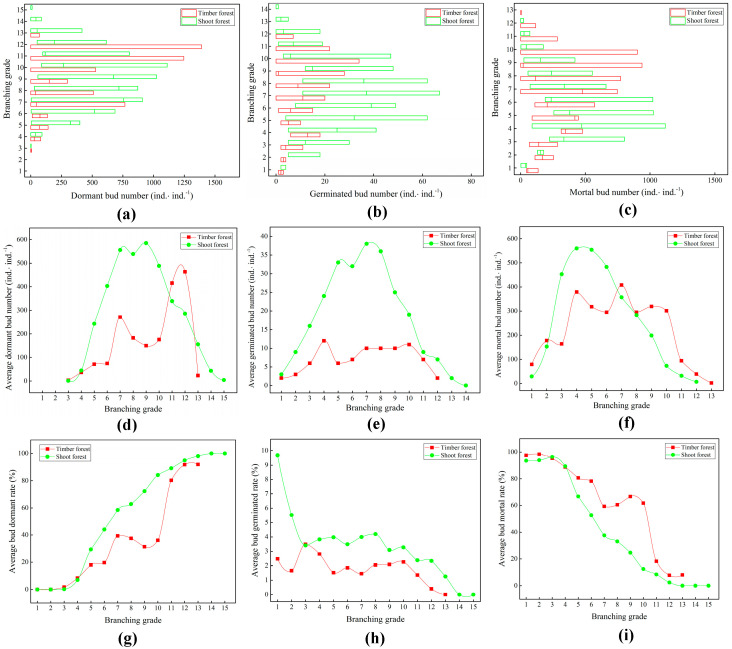
Differences in bud bank structure at different branch grades of ramet systems. (**a**) Quantity distribution of dormant buds; (**b**) quantity distribution of germinated buds; (**c**) quantity distribution of mortal buds; (**d**) change trend in average number of dormant buds; (**e**) change trend in average number of germinated buds; (**f**) change trend in average number of mortal buds; (**g**) change trend in average bud dormancy rate; (**h**) change trend in average bud germination rate; (**i**) change trend in average bud mortality rate.

Compared to the timber forest, the quantity distribution changes in the dormant buds (Figure 2a) and mortal buds (Figure 2c) of the ramet system in the shoot forest at low branch grades were relatively large, whereas those at high branch grades were relatively small. The fluctuation range of the quantity of germinated buds (Figure 2b) in each branch grade of the ramet system in the shoot forest was generally greater than that in the timber forest. This indicated that the input of the lateral buds of the underground rhizomes of the ramet system in the shoot forest was relatively stable. However, the large-scale production of bamboo shoots easily causes large-scale changes in different lateral bud types. The input of lateral buds from the underground rhizomes of the ramet system in the timber forest was relatively unstable, and the buds were prone to death. Factors in the soil environment of the timber forest were not conducive to the renewal and growth of the lateral buds of the underground rhizomes. Meanwhile, the soil environment of the shoot forest after artificial management could promote the renewal of the ramet system bud bank.

The ramet system of the shoot bamboo forest had dormant buds distributed in 13 branch grades, from 3 to 15 (Figure 2d). Meanwhile, that of the timber forest had dormant buds distributed in 11 branch grades, from 3 to 13. The peak distribution of dormant buds in the ramet system of the shoot forest was at branch grades 7–9, whereas that in the timber forest was at branch grades 11–12. The ramet system of the shoot forest had germinated buds distributed in 14 branch grades, from 1 to 14 (Figure 2e). Meanwhile, that of the timber forest had germinated buds distributed in 12 branch grades, from 1 to 12. The peak distribution of germinated buds in the ramet system of the shoot forest was in branch grades 5–8, whereas that in the timber forest was in the fourth branch grade. The ramet system of the shoot forest had mortal buds distributed in 12 branch grades, from 1 to 12 (Figure 2f). Meanwhile, that of the timber forest had mortal buds distributed in 13 branch grades, from 1 to 13. The peak distribution of mortal buds in the ramet system of the shoot forest was in branch grades 3–6, whereas that in the timber forest was in branch grades 4–10.

The average number of germinated buds for each branch grade of the ramet system in the shoot forest (Figure 2e) was generally higher than that in the timber forest. The average numbers of dormant buds (Figure 2d) and mortal buds (Figure 2f) in the middle- and low branch grades of the ramet system were relatively high. Meanwhile, those in the high branch grades of the ramet system in the timber forest were relatively high. Branches with a high number of dormant buds also had a high number of mortal buds. This indicates that the bud bank of the ramet system is mainly composed of dormant buds and mortal buds. However, this also indicates that if dormant buds do not germinate, they eventually die. The dormancy (Figure 2g) and germination rates (Figure 2h) of the lateral buds of the underground rhizomes of the ramet system in the shoot forest were generally higher than those in the timber forest. Meanwhile, the mortality rate (Figure 2i) of the lateral buds was generally lower than that in the timber forest. The vitality and productivity of the ramet system of the shoot bamboo forest are higher than those of the timber forest.

### 2.3. Branch Growth of Bamboo Ramet Systems

**Figure 3:**
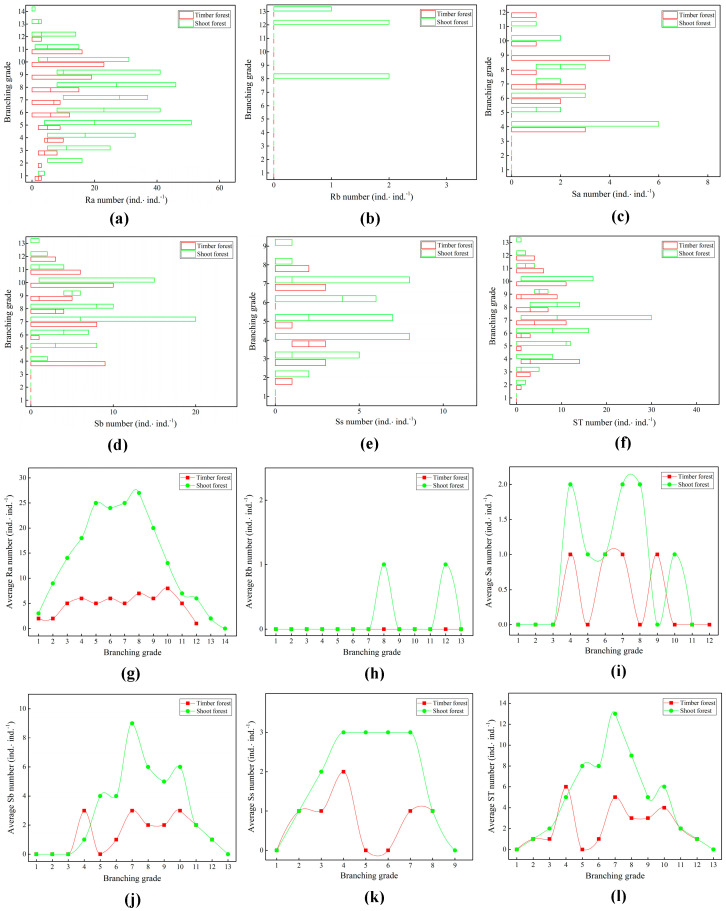
Differences in branches at different branch grades of ramet systems. (**a**) Quantity distribution of bamboo rhizomes (Ra); (**b**) quantity distribution of rhizome buds (Rb); (**c**) quantity distribution of bamboo stands (Sa); (**d**) quantity distribution of bamboo shoot buds (Sb); (**e**) quantity distribution of bamboo stumps (Ss); (**f**) quantity distribution of total bamboo shoot branches (ST); (**g**) change trend in average bamboo rhizomes; (**h**) change trend in average rhizome buds; (**i**) change trend in average bamboo standings; (**j**) change trend in average bamboo shoot buds; (**k**) change trend in average bamboo stumps; (**l**) change trend in average total bamboo shoot branches.

The variation ranges of the different branch types (Figure 3a–f) of the ramet system in the shoot bamboo forest were greater than those in the timber forest. This is related to the differential growth of different branch types stimulated by bamboo shoot harvesting in the shoot forests. The maximum number of underground bamboo rhizome (Ra) branches at different branch grades of the ramet system in the shoot bamboo forest was 27 (Figure 3g). The average total number of Ra branches grown in each ramet system was 191. The maximum number of aboveground branches of different branch grades was 13 (Figure 3l), and the average total number of aboveground branches grown in each ramet system was 61. The maximum number of underground Ra branches at different branch grades of the ramet system in the timber forest was eight (Figure 3g), and the average total number of Ra branches grown by each ramet system was 59. The maximum number of aboveground branches at different branch grades was six (Figure 3l), and the average total number of aboveground branches grown by each ramet system was 28. The branches of the ramet systems in the different management-type bamboo forests were mainly underground. The numbers of underground and aboveground branches in the shoot forest were higher than those in the timber forest.

The number of rhizome bud (Rb) branches in the ramet system during the study period was relatively small (Figure 3h). There were only individuals from the eighth and twelfth branch grades from the ramet system in the shoot forest. In both the timber and the shoot forest, the aboveground branches of their bamboo ramet systems—that is, bamboo stands (Sa), shoot buds (Sb), and bamboo stumps (Ss)—were distributed in the middle branch grades of the ramet system (Figure 3i–k) and showed a wavy change trend. This could be related to the alternate harvesting year management method for bamboo forests. In this context, in the first rotation year, many bamboo shoots and bamboo materials are harvested. The following year, relatively few or no bamboo shoots and bamboo materials are harvested, and this cycle is then repeated.

### 2.4. Branch Distribution Patterns in Bamboo Ramet Systems

**Figure 4:**
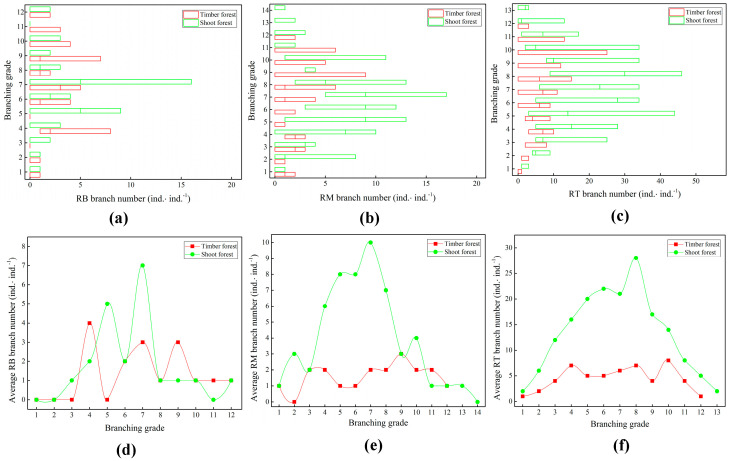
Differences in branch distributions at different branch grades of ramet systems. (**a**) Quantity distribution of branches at rhizome base (RB); (**b**): quantity distribution of branches in middle of rhizome (RM); (**c**): quantity distribution of branches at rhizome tip (RT); (**d**): change trend in average branches at rhizome base; (**e**): change trend in average branches in middle of rhizome; (**f**): change trend in average branches at rhizome tip.

The variation in branch distribution in the shoot forest was greater than that in the timber forest (Figure 4a–c). Among them, the maximum number of branches at different branch grades of the ramet system in the shoot forest was 46, and the maximum number of branches in the timber forest was 25. Both were distributed on the rhizome tip (RT) (Figure 4c). The maximum numbers of branch distributions in the rhizome base (RB) and middle of the rhizome (RM) of the ramet system in the shoot forest were 16 and 17, respectively (Figure 4a,b). Meanwhile, those in the timber forest were 8 and 9, respectively, with a relatively large difference. The branch distribution in the RB at different branch grades of the ramet system showed a clear wavy trend (Figure 4d). The maximum average number of branches distributed in each branch grade of the ramet system in the shoot forest was seven, and the average total number of branches distributed in each ramet system was 23. The maximum average number of branches distributed in each branch grade of the ramet system in the timber forest was four, and the average total number of branches distributed of each ramet system was 15.

The number of branches distributed in the RM and RT of each branch grade of the ramet system in the shoot forest was generally higher than that in the timber forest (Figure 4e,f). The average maximum number of branches distributed in the RM of each branch grade of the shoot forest was 10 (Figure 4e). The average total number of branches distributed in each ramet system was 55. The average maximum number of branches distributed in the RT was 28 (Figure 4f), and the total average number of branches distributed in each ramet system was 174. The average maximum number of branches distributed on the RM at each branch grade of the timber forest was three (Figure 4e). The average total number of branches distributed in each ramet system was 18. The average maximum number of branches distributed at the RT was eight (Figure 4f), and the average total number of branch distributions of each ramet system was 54. There were branches distributed in each part of the underground rhizome of the bamboo ramet system. However, there were substantial differences in the number of branch distributions in the different areas. The number of branches distributed at the RT was the highest, followed by that in the RM and RB.

## 3. Discussion

### 3.1. Influence of Different Management Methods on Underground Rhizome Morphology of Ramet Systems

Clonal plants can adjust their ramet systems flexibly to adapt to variable environments [18,19]. In this study, the underground rhizome morphology of the bamboo ramet systems differed between the timber and shoot forests of Moso bamboo, likely due to notable differences in management methods. During the dynamic processes of underground rhizome renewal and the growth of the ramet system, the performance of the shoot forest in the middle and low branch grades was unstable. Meanwhile, that in the high branch grades was relatively stable. In contrast, the opposite trend was observed in the timber forest, which presented relatively unstable growth in the early stages but relative stability in the later stages of aging. This is consistent with the theory that clonal plants dynamically adjust the growth patterns of their underground rhizomes according to environmental conditions [20,21,22]. In the bamboo shoot forests, measures such as frequent bamboo shoot harvesting, soil turning, and cleaning the old bamboo rhizomes and stumps make the bamboo forest soil environment relatively homogeneous [14]. This can also lead to differences in the branch morphology of the low branch grades of the ramet system. Unlike shoot forests, soil in timber forests is disturbed less by humans. The underground rhizomes of the ramet system may encounter obstructions that hinder the growth of bamboo rhizomes during their growth in the soil, resulting in the unstable renewal growth of the ramet system.

The soil environment of the timber forest retains more natural attributes. Therefore, its nutrient distribution has a high level of heterogeneity. This makes the bamboo rhizome branches of the ramet system short and thick, enhancing the stability of their structure. This is similar to the conservative survival mode of clonal plants in adverse environments that focus on consolidating their growth [23,24,25]. In contrast, through regular artificial reclamation, strategic fertilization, and other fine-scale management measures, the shoot forest creates a more suitable soil environment for bamboo growth, promoting the development of longer and thinner bamboo rhizome branches in the ramet system, and actively grows in all directions to obtain more resources. This is an active, open survival mode [26,27]. These differences indicate that different management methods, such as shaping hands and determining growth direction, and the morphology of the underground rhizomes affect ramet system strategies for resource acquisition and use.

### 3.2. Dynamic Relationship Between Bud Bank Structure and Ramet System Renewal

The bud bank is a core element for maintaining the stability and sustainable development of clonal plant populations [28] and presents substantial structural differences in different environments and under human interference patterns [29,30]. Benefiting from artificial management interventions, the input of the lateral buds of the underground rhizomes of the bamboo ramet system in the shoot forest was relatively stable. However, frequent bamboo shoot harvesting disrupts the original balance, leading to substantial fluctuations in the number of different lateral bud types. In the management of shoot forests, a suitable harvesting intensity may help maintain a certain vitality of the bud bank, but excessive harvesting may negatively impact the bud bank [31]. In contrast, in the timber forest soil environment, many factors are not conducive to the renewal and growth of lateral buds in the underground rhizomes of the ramet system, such as old bamboo stumps, stones, and water-filled pits. These obstacles may render the input of lateral buds unstable and prone to death. This may also be caused by the long-term lack of maintenance of the bamboo forest soil environment. This leads to changes in the structure of the soil microbial community and the heterogeneity of soil fertility. We found that the shoot forest effectively promoted the renewal of the bud bank through management, substantially enhancing the vitality and productivity of the ramet system.

From the perspective of the types and distribution of lateral buds, there were differences in the distribution ranges and peak numbers of dormant, germinated, and mortal buds between the shoot and timber forest bamboo ramet systems. The peak distribution of dormant buds in the shoot forest occurred at branch grades 7–9, whereas it occurred at branch grades 11–12 in the timber forest. This shows that different management methods profoundly change the dynamic balance of the bud bank of bamboo ramet systems. Plant bud banks largely determine population renewal and community construction [32,33]. Therefore, dynamic changes in bud banks have a profound effect on the renewal and growth of ramet systems. During the dynamic evolution process of the ramet system, the state transition of lateral buds on the underground rhizome plays a decisive role in the development of the ramet system. However, if dormant buds do not have the opportunity to germinate, they are likely to die. This was observed in both types of managed bamboo forests.

### 3.3. Responses of Branch Growth and Distribution of Ramet Systems to Artificial Management

Different environments and human interference methods can guide the branch growth and distribution patterns of clonal plants in different directions [34,35]. This is also evident in bamboo plants [36,37]. Frequent bamboo shoot harvesting strongly stimulates branch growth in the ramet system, resulting in greater variation in branch types in shoot forests than in timber forests. The number of underground and aboveground branches is generally higher in shoot forests than in timber forests. The bamboo shoot harvesting in the shoot forest breaks the original growth balance of the bamboo. Under certain conditions, plants make physiological adjustments to maintain growth and stimulate branch development [38]. In shoot forests with long-term, high-intensity bamboo shoot harvesting, the bamboo ramet system increases the number of underground rhizome branches in order to obtain more nutrients and space resources. This provides more possibilities for the growth of bamboo shoots. These results show the strong influence of management activities on branch growth in bamboo ramet systems.

In terms of the branch distribution pattern, in both timber and shoot forests, the aboveground branches were mainly concentrated in the middle branch grades of the ramet system and showed a wavy change trend. This was closely related to the alternate-bearing-year management method used in bamboo forests. The alternate-bearing-year phenomenon in bamboo [39] leads to differences in its nutrient requirements and distribution in different years [40,41]. In its first year, bamboo allocates more nutrients to the growth and development of bamboo shoots. In the second year, it shifts focus to the vegetative growth of rhizomes and nutrient storage. This periodic redistribution of nutrients may influence branch growth and distribution in bamboo ramet systems. There were significant differences in the number of branches in different parts of the bamboo ramet system. The number of branches at the RT was the largest, followed by those in the RM and RB. The variation in branch distribution in the shoot forest was greater. Many complex factors may be influencing this, such as differences in the growth and differentiation of lateral buds on the underground rhizome, differences in growth vitality in different parts, the uneven distribution of nutrients, and different degrees of interference from management activities on the underground rhizomes.

This study provides valuable insights into how the morphological, structural, and functional characteristics of the ramet system in Moso bamboo change under different management practices. However, to deeply understand the regulatory mechanisms governing the development and growth of the bamboo ramet system, further exploration is required. First, the regulation of endogenous hormones in the bamboo ramet system (such as auxin and cytokinin dynamics) must be analyzed and the processes underlying nutrient storage, transportation, and clonal integration must be assessed. Such work is crucial for understanding the mechanistic basis of the observed phenotypic plasticity. Second, the direct causal relationship between soil characteristics in bamboo forests and ramet system dynamics must be investigated and analyzed in order to quantify the nutrient allocation within the ramet system and its effects on rhizome growth and lateral bud activation through isotope labeling. Third, long-term fixed-position monitoring of the growth and dynamic changes in the bamboo ramet system must be implemented to accurately capture the adaptive strategies of the ramet system under long-term anthropogenic disturbances or environmental changes. Finally, multi-omics analyses (such as transcriptomics and metabolomics) of lateral bud differentiation and branching development in the rhizomes of bamboo ramet systems are required to further elucidate the epigenetic regulatory mechanisms behind the phenotype changes induced by different factors.

## 4. Materials and Methods

### 4.1. Experimental Site

**Figure 5:**
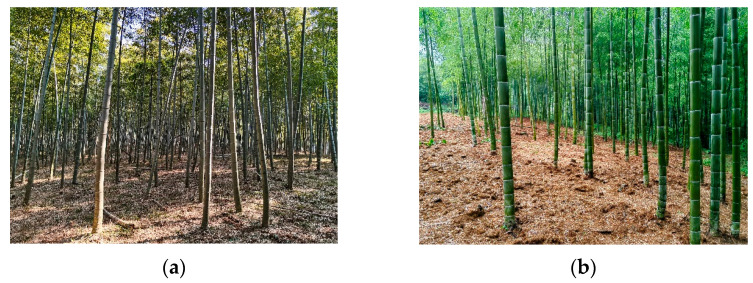
Different management types of bamboo forests. (**a**) Timber bamboo forest; (**b**) shoot bamboo forest.

The Moso bamboo forest experimental base was located in Huizhouzhuang Village (119.87° E, 31.12° N), Shuikou Township, Changxing County, Huzhou City, Zhejiang Province, China. The study area had a subtropical monsoon climate, with an average annual precipitation of 1309 mm, an average annual temperature of 15.6 °C, and an average annual sunshine duration of 1810 h. It was adjacent to Taihu Lake in the east and had flat terrain, fertile soil, and sufficient water sources for bamboo growth. In a continuous bamboo forest area of more than 30 ha, bamboo forests on a gentle slope and flat ground on the sunny side were selected as the sample plots. The areas under timber and shoot bamboo forest management, the two management types being investigated, were both more than 1 ha (Figure 5). Moso bamboo forest management involves large and small harvests in alternate years. In the first year of the bamboo forest harvesting cycle, the harvest of bamboo shoots and bamboo materials is relatively large. Meanwhile, in the second year of the harvesting cycle, there is a relatively small harvest. This is caused by the long-term application of artificial management methods. In a closed adult bamboo forest, the “degree” refers to the sum of the first and second years of the harvesting cycle; that is, two years is one degree.

The underground component of the timber forest had no soil turning, fertilization, or bamboo shoot harvesting. The soil environment and growth of underground bamboo rhizomes are rarely disturbed by humans. In the aboveground part of a timber forest, bamboo stands are naturally preserved from bamboo shoots, and bamboo materials are harvested strategically. The underground part of the shoot forest was reclaimed once every two to three years. At the end of autumn, every two years, organic fertilizer for bamboo shoot growth, such as 2–3 t/ha of decomposed duck manure fertilizer, was applied, and the soil was shallowly turned. Three types of bamboo shoots were harvested, namely, spring bamboo shoots and rhizome tips, that is, edible bamboo shoots in summer and winter bamboo shoots. The soil environment and growth of underground bamboo rhizomes are often subject to disturbance by humans. For the aboveground parts of the shoot forest, bamboo stands were preserved through artificial selection and bamboo materials were harvested strategically. Owing to the long-term differences in bamboo forest management methods, there were differences in bamboo forest soil and bamboo growth levels (Table 1).

### 4.2. Investigation and Analysis

#### 4.2.1. Excavating Ramet Systems by Opening Exposed Rhizome Pits

**Figure 6:**
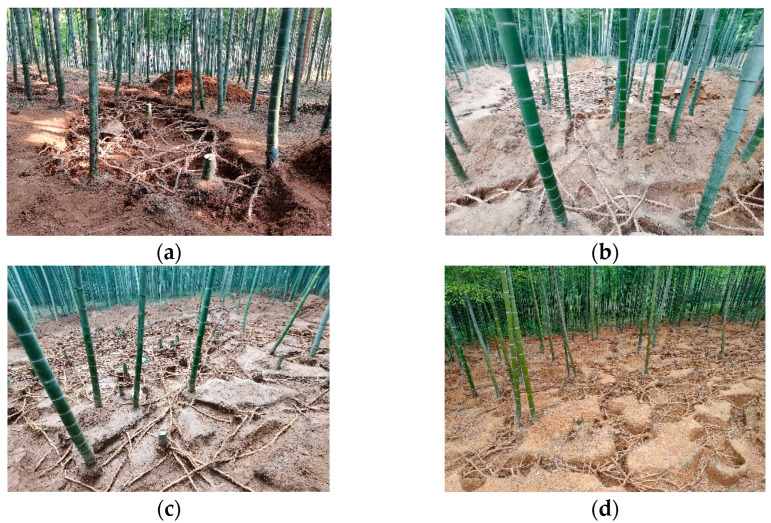
Investigation of bamboo ramet systems. (**a**) Opening soil windows; (**b**) combing bamboo rhizomes; (**c**) ramet system of timber bamboo forest; (**d**) ramet system of shoot bamboo forest.

The excavation and investigation of the bamboo ramet system were based on the re-search methods of Gao et al. [14], with appropriate adjustments made according to specific circumstances (Figure S1). In the central area of the bamboo forest, that is, not at the forest edge, exposed rhizome pits were randomly dug on-site (Figure 6a). The soil surface of the exposed rhizome pit was cleared until the bamboo rhizome was visible. Then soil was excavated layer-by-layer and carefully cleaned. The bamboo rhizomes were not cut in order to maintain the integrity of the interconnected structure. The size of the exposed rhizome pit was then gradually increased. The bamboo standing within the exposed rhizome pit was cut at a height of 20–30 cm from the ground, and the bamboo roots were cut off to make the connection between the bamboo stands and the bamboo rhizomes visible. The size of the open exposed rhizome pit was more than 10 × 10 m to allow for the investigation of the growth structure of the ramet system. After the exposed rhizome pit had been dug, we randomly selected a healthy bamboo rhizome with an average diameter from the bamboo rhizomes in the exposed rhizome pit and excavated a complete bamboo ramet system comprising three replicates. Along the growth direction and the opposite direction of the lateral buds of the selected bamboo rhizome, all the bamboo stands were excavated and cut 20–30 cm from the ground, and the bamboo rhizomes that were connected to it were dug out (Figure 6b). The entire ramet system was excavated (Figure 6c,d).

#### 4.2.2. Investigation of Branch Growth of Bamboo Ramet Systems

**Figure 7:**
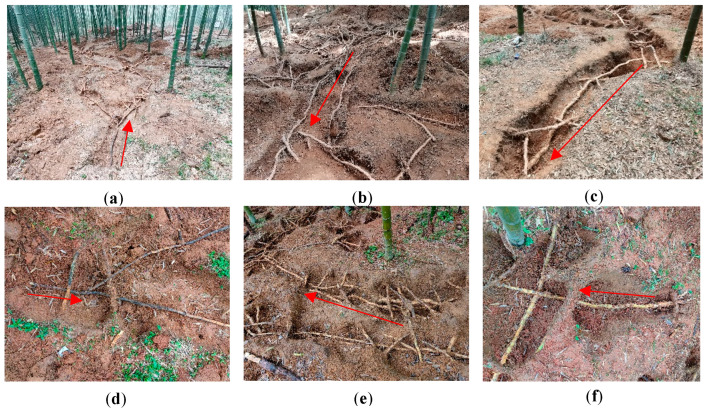
The growth structure of the bamboo ramet systems. (**a**) The initial end of the ramet system of the timber forest; (**b**) the middle part of the ramet system of the timber forest; (**c**) the growth terminal end of the ramet system of the timber forest; (**d**) the initial end of the ramet system of the shoot forest; (**e**) the middle part of the ramet system of the shoot forest; (**f**) the growth terminal end of the ramet system of the shoot forest. The direction of the arrow indicates the growth direction of the ramet system.

The length, rhizome diameter, and number of rhizome nodes of each bamboo rhizome were measured from the initial to the terminal end of the ramet system (Figure 7). The number of bamboo rhizomes (Ra), rhizome buds (Rb), bamboo stands (Sa), bamboo stumps after bamboo stands were cut (Ss), and bamboo shoot buds (Sb) germinated from the lateral buds of each bamboo rhizome was recorded, as well as the number of dormant buds and mortal buds. The dormant bud rate was the number of dormant buds/total number of buds, the germination rate was the number of germinated buds/total number of buds, and the mortality rate was the number of mortal buds/total number of buds. Meanwhile, the different branch types (Ra, Rb, Sa, Ss, and Sb) and the rhizome node locations were recorded. The bamboo rhizomes were divided into three equal parts based on the number of rhizome nodes. The part close to the rhizome base was known as the RB, the middle part was identified as RM, and the RT was located close to the tip. The distribution pattern of branches was then counted.

#### 4.2.3. Data Analysis

Microsoft Excel 2016 (Microsoft Corporation, Redmond, WA, USA) was used to calculate the average values of the data. Origin 2016 software (OriginLab Corporation, Northampton, MA, USA) was used to plot the data. Field investigations showed that the growth indicators (e.g., morphological characteristics) of each branching grade in the bamboo ramet system fluctuated significantly. This inherent variability remained even when excavating ramet systems at the same core forest position. Thus, box-and-whisker plots were used to characterize the data dispersion range.

## 5. Conclusions

Different management methods cause differences in bamboo ramet system growth. The underground rhizome renewal of the ramet system in the shoot forest was relatively stable in the early stage and unstable in the later stage. Meanwhile, the opposite was observed in the timber forest. The fine-scale management of shoot forests promotes thin and long bamboo rhizome branches in the ramet system, which is conducive to active spatial expansion. The near-natural state of the timber forest shortened and thickened the bamboo rhizome branches, focusing on consolidation. The artificial management of the shoot forest stabilizes the input of lateral buds, but bamboo shoot harvesting disturbs the bud bank balance. The heterogeneity of the soil environment in timber forests leads to the unstable input of lateral buds and their easy death. The two forest types differed significantly in their distribution ranges and peak numbers of dormant, germinated, and mortal buds. The close relationship between the bud bank and the renewal of the ramet system, that is, the fact that dormant buds will eventually die if they cannot germinate in time, provides a theoretical basis for the management of bud banks in the sustainable management of bamboo forests. Bamboo shoot harvesting has a strong stimulatory effect on the branch growth of ramet systems. The variation ranges of branch types and the number of branches in the shoot forest far exceeded those in the timber forest. These findings highlight the key role of human management activities and provide strategic guidance for improving the productivity of bamboo forests by regulating the branch growth and distribution of ramet systems in bamboo forest management. For example, growth vitality can be enhanced by streamlining the structure of the ramet system, and the branch density at the terminal end of the ramet system can be increased by reasonably harvesting rhizome tips (i.e., rhizome shoots), thereby promoting the self-renewal of the ramet system.

## Figures and Tables

**Table 1:** Bamboo forest structure and basic soil properties.

Bamboo Forest Type	Bamboo Forest Structure		Bamboo Forest Basic Soil Properties						
	Diameter at Breast Height (cm)	Standing Density (Plants/ha)	pH Value	Total Nitrogen (g/kg^−1^)	Total Phosphorus (g⋅kg^−1^)	Hydrolyzable Nitrogen (mg⋅kg^−1^)	Available Potassium (mg⋅kg^−1^)	Organic Matter (g⋅kg^−1^)	Available Phosphorus (mg⋅kg^−1^)
Timber forest	12.10 ± 1.47	3611 ± 278	4.65 ± 0.09	1.29 ± 0.18	0.33 ± 0.02	108.00 ± 13.45	33.50 ± 8.43	25.70 ± 3.80	2.02 ± 0.21
Shoot forest	11.08 ± 1.12	3014 ± 285	4.83 ± 0.05	1.28 ± 0.17	0.23 ± 0.01	129.67 ± 24.79	67.97 ± 27.33	24.67 ± 4.19	3.31 ± 1.63

## Data Availability

Data are contained within the article.

## Supplementary Materials

The following supporting information can be downloaded at: https://www.mdpi.com/article/10.3390/plants14121835/s1, Figure S1: Schematic diagram of bamboo ramet system.
