# Supplementary material for: Effects of Different Management Practices on Ramet System Dynamics in Moso Bamboo (Phyllostachys edulis) Forests, China
Source: Plants (Basel). 2025 Jun 14;14(12):1835. doi: 10.3390/plants14121835 (PMC12196740; doi:10.3390/plants14121835)
Supplement: Supplementary file 1 [file plants-14-01835-s001.zip › plants-3684564-supplementary.pdf]

Supplementary materials

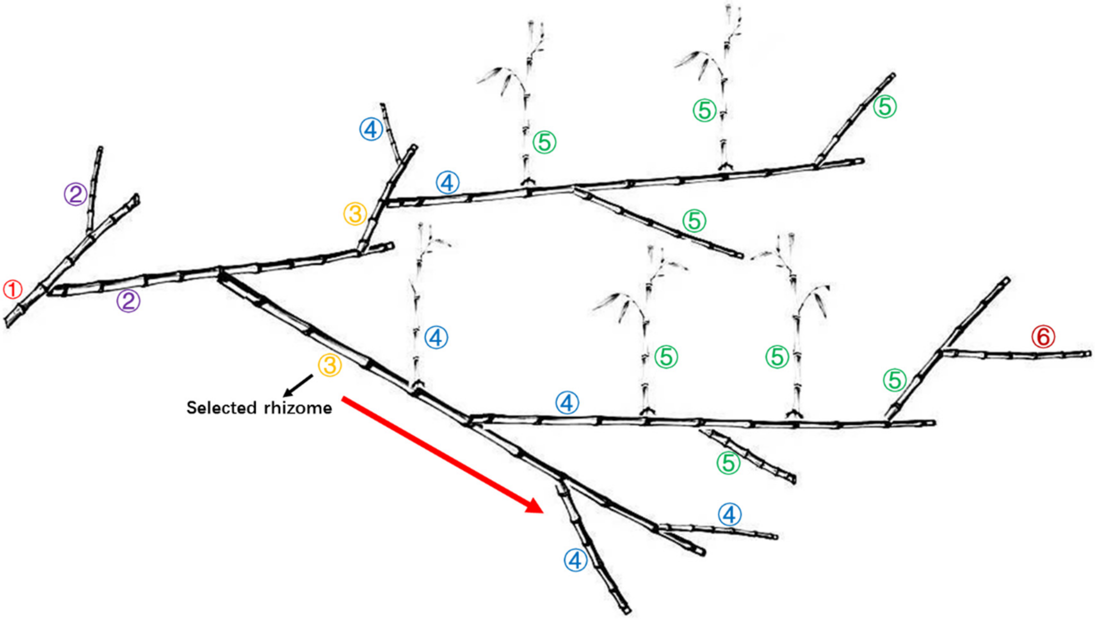

Figure S1. Schematic diagram of the bamboo ramet system. The same number indicates the same branching grade. When excavating the ramet system, take the 3rd-grade bamboo rhizome (as indicated by the black arrow) as the selected rhizome for example. First, along the growth direction of the buds on the rhizome (as indicated by the red arrow), sequentially excavate each connected branch until reaching the end. Then excavate along the opposite direction of the lateral buds' growth on the rhizome. When excavating to the connected 2nd-grade bamboo rhizome, sequentially excavate each connected branch along the growth direction of the lateral buds of the 2nd-grade rhizome until reaching the end. Then excavate along the opposite direction of the lateral buds' growth on the 2nd-grade rhizome. Proceed in this manner until the complete ramet system is excavated. When counting each index, start from the 1st-grade bamboo rhizome and sequentially count and survey along the growth direction of the lateral buds.
